# Features of the influence of hereditary factors on the clinical manifestations of depressive disorders

**DOI:** 10.1192/j.eurpsy.2021.888

**Published:** 2021-08-13

**Authors:** N. Maruta, S. Kolyadko, V. Fedchenko, I. Yavdak

**Affiliations:** Borderline Psychiatry, “Institute of Neurology, Psychiatry and Narcology of NAMS of Ukraine” SI, Kharkiv, Ukraine

**Keywords:** depression, hereditary factors

## Abstract

**Introduction:**

The urgency of the problem of depression is due to their high prevalence and severity of consequences. At present, the pathogenetic role of heredity in the course of depressive disorders remains unclear. Therefore, studies related to this problem are designed to identify the relationship between hereditary factors and the characteristics of the clinic of depression.

**Objectives:**

The aim was to study the features of the influence of hereditary factors on the clinic of depressive disorders.

**Methods:**

clinical-psychopathological, psychometric, genealogical, statistical.

**Results:**

Based on the study of clinical, psychometric (Hamilton scale (HDRS)), genealogical data of 87 patients with depression, a high level of family burden of depression at all levels of kinship in the pedigree of patients (73.56%), alcohol abuse (39.08%), the presence of hypertension (54.02%), heart disease (42.53%) and endocrine pathology (14.94%) were identified. Moreover, in the pedigree of the examined most often this pathology was found in relatives of I and II degree of kinship. When comparing the factors of heredity with the clinical structure and features of depression revealed the proportion of correlations of such factors as: observation by a psychiatrist of I and II degree of relatedness (p ≤ 0.01), depressive disorders mainly by II degree of relatedness (p ≤ 0.05), suicidal behavior according to I and II degree of kinship (p ≤ 0.005), alcohol dependence mainly on I degree of kinship (p ≤ 0.03). Selected leading symptom complexes: depressive, asthenic, apathetic, anxiety-phobic, somato-vegetative, hypochondriac.

**Conclusions:**

The data obtained should be taken into account in diagnostic and preventive measures.

